# Round the Hospitals

**Published:** 1919-04-05

**Authors:** 


### ROUND THE HOSPITALS.

The King held an Investiture on March 27, in the ball-room of Buckingham Palace, and personally conferred the Royal Red Cross, Second Class, on members of the nursing service as follows: Sister Elsie King, T.F.N.S.,; Sister Clare Murray and Sister Harriet Rainbow, Civil N.S.; Sister Elizabeth Smith, B.R.C.S.; Sister Mary Fisher and Sister Wilfred Scott, Australian N.S. Also on Miss Florence Abell, V.A.D. Queen Alexandra received these ladies at Marlborough House subsequent to the Investiture.

At the Investiture held in the Ball-room of Buckingham Palace on March 29, the King personally conferred the decoration of the Royal Red Cross Second Class on Staff Nurse Christina Oliver, Q.A.I.M.N.S.R.; on Matron Margaret O'Neill and Sister Sybil Harry, B.R.C.S.; on Matron Lucy Thompson and Sister Sarah Chadwick, St. John's A.B.; and on Miss Jessie Slocomb and Miss Katharine Wyld, V.A.D. These ladies had the honour of being received subsequently by Queen Alexandra at Marlborough House.

**ROUND THE HOSPITALS—(*continued*).**

The Queen paid a visit to Queen Charlotte's Hospital on March 27, leaving every one cheered and happy. The sisters formed a guard of honour in the hospital forecourt, and Her Majesty paused to take special notice of those who wore war decorations and to inquire about their services. Her interest in babies was never more delightfully manifested; and a French patient whose husband is reported missing was comforted by a little sympathetic talk in her own tongue. The hospital needs £40,000 for extension and improvement, and the Queen expressed hearty approval of the appeal and of the excellent work carried on in the institution.

Queen Alexandra, as President of the British Red Cross Society, has presented to the Queen of Roumania the gold medal of the Society in recognition of her war work. The medal bears the inscription: "To her Majesty the Queen of Roumania, in recognition of her devoted and unselfish services in the relief of suffering during the war 1914-1919." Queen Alexandra said the British Red Cross Society was anxious to show its high appreciation of the splendid services the Queen of Roumania had rendered to her subjects who had suffered so much in the war, and also wished to express their gratitude to her for innumerable acts of kindness shown by her to the members of the Society who had worked in Roumania. The Queen of Roumania spoke warmly of the help given by the Red Cross, specially in the early stages of the war.

The published will of Miss Luckes, C.B.E., R.R.C., shows that the late Matron of the London Hospital left property of the gross value of £4,178, with net personalty £3,804. To Mr. Ernest W. Morris, Secretary of the hospital, is left £100 "as a token of my affectionate appreciation of his sympathetic friendship and practical help in my work." £200 is bequeathed to Dr. Theodore H. Thompson "in grateful remembrance of his skill and unfailing attention which saved my life in my very serious illness in 1916," and "for the kindness with which I know he will watch over me until the end." To her assistants, Ethel Annie Sandifer and Janet Scott, £100 each is left, and £50 each to five others "in grateful remembrance of loyal and affectionate service and of our happy work together." Lord Knutsford, Chairman of the Hospital, receives two bequests—a gold and jewelled pen and £100 to purchase a picture or some other keepsake" in memory of a pleasant friendship." The sum of £200 is set aside for a portrait or bust of Lord Knutsford to be placed in the Nurses' sitting-room in the Home, which has been named after Miss Luckes, to remind future generations of nurses of Lord Knutsford's efforts to establish the Home. The two head porters are each left £10 for "all they have done for me since I needed so much personal care and attention." The residue of the property goes to Miss Thyra Larsen, one of Miss Luckes' assistants, "in grateful and affectionate remembrance of her kind care of me and of her perfect loyalty and invaluable help in my work."

We learn with pleasure from the sister-matron, Miss M. A. Willcox, that a considerable increase has taken place in the salaries of the ward sisters at King's College Hospital. They now commence at £50, rising £10 annually to £80. The course of training for probationers has been extended to four years, the salary being £10, £15, £20, and £30 in the respective years. Under this arrangement V.A.D. members who have done two years' consecutive work in a recognised military hospital will be eligible for a three years' course, the fourth year of training being omitted. In its new scale of salaries King's College Hospital sets a good example in making the sisters' posts really advantageous. First-year probationers at this hospital, it may be mentioned, formerly received no salary.

The committee of the Coventry and Warwickshire Hospital has adopted a scheme drawn up by the matron, Miss S. Hutchinson, whereby the staff not only have one full day off in seven, but also increased off-duty time each day. Under this admirable time-table the sisters and staff nurses have four hours off duty every day, 1 to 5 p.m., and 6 to 10 p.m. alternately, with extra privileges on Sunday. The probationers have also four hours daily, 9 to 1 p.m. 1 to 5 p.m., and 6 to 10 p.m. alternately. Night nurses go off duty at 7.30 instead of 8.30 a.m. They will also have a clear hour out of the wards some time between 12 and 3 a.m., going to the dining-room, where an attendant has been put on night duty to see to their comfort, for their principal meal. All lectures are to be given to nurses during duty hours. The matron has had the sanction of her committee to keep an extra staff for holiday duty. The staff, which now totals seventy-nine, will undergo considerable expansion. This is in many respects the most modern and thoroughly satisfactory scheme so far brought out.

The question of pensioning institution nurses has been engaging the attention of the committee of King Edward's Hospital Fund. A small sub-committee appointed in 1914 to inquire into the subject of staff-pensions in hospitals has just reported. They recommend that every hospital nurse before completing her training should be assisted to initiate some provision for old age. This is already done, with excellent results, in many institutions. But something more than this is needed to ensure the future of the women who give their lives to institution work as matrons or heads of departments, and the system of staff pensions on a contributory basis outlined by the sub-committee should prove of great value.

The St. Pancras Infirmary Committee has recommended the board to grant three days extra leave to the nursing staff this year, as some recompense for the extra duties and strain imposed upon them during the recent epidemics. Originally, the committee advised the board to grant small money gratuities to the nursing staff, but this was vetoed when the recommendation came up at the board meeting. However, the board has relented

**ROUND THE HOSPITALS—(*continued*).**

so far as to grant the recommendation, now made, for extra leave.

THE committee of the Salisbury Infirmary (County Hospital) has decided to grant the nursing staff one day off duty in each week as soon as the staff can be sufficiently increased. Salaries have now been raised to £15, £20, £25 (with bonus of £5 on completion of training), and £40. Ward sisters £50 to £60; sisters of special departments £55 to £65; home sister and night superintendent £60 to £70. We congratulate Miss Cable on this admirable advance, and feel sure she will soon obtain the additional pupil nurses she needs.

THE committee of the Stockport General Infirmary has unanimously approved a scheme to enable the staff to have one day off in seven, and has authorised the matron, Miss Goodacre, to engage the necessary number of extra nurses as soon as possible. An additional sister much needed to ensure the smooth working of the institution is to be engaged. The sisters now in office are to have their salaries raised to £55, rising by £5 increment to £65. Newly appointed sisters start at £50 and rise to £65. Probationers' salaries were recently raised to £14, £17, £20, with made-up uniform. A proposal is before the committee to grant free midwifery training and entrance fee to the C.M.B. examination to the top nurse in the Final Examination. Miss Goodacre will be glad to hear of ladies wishing to become probationers, in order to bring her staff up to full strength.

NEW regulations for probationers at the Royal Hampshire County Hospital, Winchester, have been in force some weeks and are proving a great success. The matron, Miss Turner, with the assent of her committee has arranged a time-table which allows for four hours off-duty every day, 7 to 11, or 10 to 2, or 2 to 6, or 5 to 9. This good spell off-duty every day is much appreciated by the nurses. The morning time, from 7 to 11, is specially enjoyed as it means the luxury of an extra hour in bed and a good walk before coming on duty. The nurses have on Sunday, alternately 10 to 2 or 2 to 8.30. They have also one whole day a month from 6 p.m. the previous day, and those on night duty have two nights off monthly. Junior nurses at present have three weeks holiday in the year but it is hoped soon to increase this. Salaries are £14, £20, and £26. Fourth year nurses who have passed their examinations and gained certificates receive £40, the others £30.

AT the York County Hospital the Committee has arranged to increase the annual holiday of nurses in training from three weeks to one month. Nurses are from now on to have three hours off duty daily, with a half-day weekly and a long day monthly. The night nurses are to have a long evening weekly and four nights off duty in the twelve weeks. This new arrangement will shorten the nurses' working week by 10½ hours, a very considerable improvement.

THE promoters of the Nurses' Home at St. Bartholomew's have issued an appeal to each of the 300 sisters and nurses on the staff to collect £3 for the fund. Every governor is being asked to make himself responsible for the cost of one nurse's accommodation, this being reckoned at £350. If all do their part the whole amount will speedily be raised. We are sure that the nurses and doctors who have left the great hospital and carried its traditions into every part of the world will not rest satisfied until they have contributed to the long-anticipated Nurses' Home. The hon. secretary of the fund will no doubt cast his net far and wide. Wherever there is a Bart's man or woman he will surely find response.

IT is good news and will cause immense relief in many quarters to hear that the Army Council has decided to modify the conditions for the release of members of Q.A.I.M.N.S.R., the T.F.N.S. and assistant nurses, as well as for V.A.D. nursing members and special probationers serving in military hospitals. Under Army Council Instruction 205 it has been decided that from the date of issue any member of the above services wishing to do so may submit an application for early release through the usual channels of the War Office. If the application be approved and the nurse can be spared she will be released forthwith without forfeiting any "financial benefits" due to her. If the resignation take place without the approval of the War Office in the case of a nurse who has signed on to serve for as long as required, she naturally not only forfeits the gratuity to her, but will be required to refund a proportionate amount of the additional pay bestowed in virtue of her agreement.

WE learn that the Appointment Inquiry Bureau of the College of Nursing is working at high pressure in these days under the temporary charge of Miss Jolley, A.R.R.C. It was fully to be expected that demobilised nursing sisters after their long war service would display a great craving for appointments which enable them to enjoy home freedom and interests. The spheres of school nursing, health visiting, welfare work, and the like are attracting many, but inevitable delay is caused in taking up such appointments, not from lack of vacancies, but on account of the special certificates and courses of training which they usually entail. Sisters awaiting demobilisation could turn the waiting period to good account by making arrangements to procure these qualifications at the first moment after being set free so that no time is wasted.

INFORMATION has reached us that an order has recently been posted up at the New End Military Hospital, Hampstead, warning the sisters that they will be held absolutely responsible for all losses in the linen-room; and that the inventory will be taken every month. We confess it does not sound to us an equitable decision. It is an easy assumption that when articles are missing it is the sister's fault, but it is in the experience of most people that certain military patients have a magpie faculty for impounding articles of raiment, and old cam-

**ROUND THE HOSPITALS—(*continued*).**

paigners who have had to fend for themselves under stress of war are apt to think very little of the transgression. Sisters ought not to be mulcted of their earnings because while exercising reasonable care they find themselves outwitted.

The Health Committee of Swansea has provoked a dispute with the Swansea Nursing Association, and its action has not been tactful. This particular Health Committee seems to have broken all the unwritten rules which govern intercourse between one body and another. It seems that the Swansea Nursing Association applied for a grant from the American Red Cross Society in order to establish a maternity centre. A grant of £2,000 was made, and according to the usual procedure was allocated through the Health Committee, which, ignoring the Nursing Association altogether, decided to impound the whole sum for a municipal project of its own. The Nursing Association Committee, justly indignant at being ignored after its twenty-five years' work in the town, has resigned, and we understand the Association has been actually dissolved. There is some hope that the Mayor may take strong action, and the Health Committee brought to its senses.

At the meeting held on March 27, under the auspices of the committee of the British Women's Hospital, at the Royal Automobile Club, Pall Mall, Sir Arthur Stanley outlined the means which are to be taken to complete "The Nation's Fund and Tribute to our Nurses." The movement has already met with a cordial reception in every part of the Kingdom. It remains for London to put the finishing touch. The week beginning April 29, will be "The Nurses Tribute Week." On May 1, 2, and 3, a bazaar will be held in the fore-court of Devonshire House and all the principal hospitals in London will have representatives at the stalls. The various branches of the British Red Cross Society throughout the kingdom are being invited to co-operate and are showing ready acquiescence. The Flag Day will be held under the patronage of Queen Alexandra, who has expressed the wish that the whole of the money collected on this day shall be used for the benefit of nurses in sickness and distress. The promoters of the movement should see to it that their publicity (Press) arrangements are made more effective.

The Central Midwives Board has just presented its report for 1918, and it proves an unusually full and instructive document. The figures relating to the numbers and occupation of the midwives on the roll are presented with a lucidity and careful analysis worthy of high commendation. It is a distressing fact that though the number of names appearing on the midwives' roll increases annually, the proportion of practising midwives diminishes. This, however, is to some extent counterbalanced by the fact that out of the 1,548 successful candidates in 1918, 845, or 54.6 per cent., declared their intention of practising as midwives, and of this number 471, or 55.7 per cent., intended to practice in rural districts. This compares well with 1917, when only 50 per cent. of the successful candidates meant to practise, and only 52.7 per cent. of these in rural districts. There were seventy-six penal cases, resulting in the removal of forty-seven women from the roll. More than one-third of these cases arose out of neglect to call in medical aid in cases of ophthalmia neonatorum.

Institutions and homes under the management of approved midwives in London and the neighbourhood having been placed under inspection by the Central Midwives Board, a certified midwife, recently a teacher of pupil midwives, has been appointed inspector. It appears that a great want exists for teachers of pupil midwives possessed of higher qualifications, and the inspector reports that a higher examination, practical and theoretical, to include methods of teaching for midwives, would raise the standard. She suggests the title of first-class midwives for this grade, which, if it can be brought into being, is likely to raise the midwifery service by introducing posts suited to women of higher education.

Sarah Hincks, aged eighty, one of Miss Florence Nightingale's nurses, and on terms of intimate friendship with her till the end, was killed in the Holloway Road on March 26 owing to being knocked down by a dog. Her right thigh was fractured and her back injured. She received every care at the Islington Infirmary to which she was removed, but died from pneumonia and congestion of the lungs. She had private means, and has lately been living in the Nurses' Home, Eden Grove, Holloway.

A Central Council for Infant and Child Welfare has now been formally inaugurated at the offices of the British Red Cross Society, 83 Pall Mall. Eleven societies have affiliated and send representatives to the Council, the objects of which are to co-ordinate the different organisations concerned in the care of motherhood, infancy, and childhood, to establish, supervise, and maintain such residential institutions as may prove to be needed in these interests, and to provide and promote a standardisation of training for social workers connected with motherhood and infancy, and raise the status and pay of the workers. The chairman of the new Council is Sir Arthur Stanley, and the secretary Miss Wilson, whose abilities have been already well tested in connection with Red Cross work at the Central Office.

☞ It is our invariable practice to pay for all items of news which we may publish. The minimum payment is 5s. Paragraphs of about twenty lines in length—that is to say, of 150 words or less—are preferred. Contributions should be addressed to The Editor, The Hospital, 28 and 29 Southampton Street, Strand, London, W.C. 2. and, to be in time for the current week's issue, should reach him by the first post on Mondays.

